# High intratumoural accumulation of stealth® liposomal doxorubicin (Caelyx®) in glioblastomas and in metastatic brain tumours

**DOI:** 10.1054/bjoc.2000.1459

**Published:** 2000-10-26

**Authors:** M I Koukourakis, S Koukouraki, I Fezoulidis, N Kelekis, G Kyrias, S Archimandritis, N Karkavitsas

**Affiliations:** Tumour and Angiogenesis Research Group, 18 Dimokratias Ave, Heraklior, Crete, 71306, Greece; Department of Radiotherapy/Oncology, Medical School, University of Thessalia, Larissa; Department of Nuclear Medicine, University Hospital of Iraklion, Iraklion, Crete, 71110; National Center for Scientific Research, ‘Demokritos’, 15310 Ag. Paraskevi, Athens, Greece

**Keywords:** brain metastasis, glioblastoma, stealth liposomal doxorubicin, radiotherapy

## Abstract

The blood–brain barrier is a major obstacle for the chemotherapeutic drugs to effectively reach primary or secondary brain tumours. Stealth® liposomal drugs are highly accumulated in tumoural tissues. In the present study we investigated the relative accumulation of^99m^Tc-DTPA radiolabelled stealth® liposomal doxorubicin (Caelyx®) in 10 patients with metastatic brain tumours and five patients with brain glioblastoma undergoing radiotherapy. Patients with metastatic brain lesions were treated with 10 consecutive fractions of radiotherapy (whole brain, 3 Gy/fraction, day 1–12) followed by a booster dose of 9 Gy (3 Gy/fraction, day 21–23). Caelyx®, at a dose of 25 mg mg^–2^was given on day 1 and on day 21. Radiolabelled Caelyx® accumulation was 13–19 times higher in the glioblastomas and 7–13 times higher in the metastatic lesions, as compared to the normal brain. The drug accumulation in the tumoural areas was 40–60% of the accumulation in the bone marrow of the skull bones. The normal brain radioactivity was <4% of the bone marrow, confirming an important shielding effect of the blood–brain barrier in the normal but not in the tumoural tissue. Four of 10 patients with metastatic lesions showed a complete response in CT-scan performed 2 months following therapy. There was no severe toxicity related to radiotherapy or to chemotherapy noted. It is concluded that stealth® liposomal drugs selectively overcome the blood–brain barrier in the tumoural areas. The clinical importance of this observation is now under investigation. © 2000 Cancer Research Campaign


British Journal of Cancer (2000) **83**(10), 1281–1286
© 2000 Cancer Research Campaign
doi: 10.1054/ bjoc.2000.1459, available online at http://www.idealibrary.com on IDEAL®

### High intratumoural accumulation of stealth® liposomal doxorubicin (Caelyx®) in glioblastomas and in metastatic brain tumours

**MI Koukourakis[1,2], S Koukouraki[1,3], I Fezoulidis[2], N Kelekis[2], G Kyrias[2], S Archimandritis[4] and N Karkavitsas[3]**

[1]Tumour and Angiogenesis Research Group, 18 Dimokratias Ave, Heraklior 71306, Crete, Greece; [2]Department of Radiotherapy/Oncology, Medical School, University of Thessalia, Larissa; [3]Department of Nuclear Medicine, University Hospital of Iraklion, Iraklion 71110, Crete; [4]National Center for Scientific Research, 'Demokritos', 15310 Ag. Paraskevi, Athens, Greece

**Summary** The blood–brain barrier is a major obstacle for the chemotherapeutic drugs to effectively reach primary or secondary brain tumours. Stealth® liposomal drugs are highly accumulated in tumoural tissues. In the present study we investigated the relative accumulation of $^{99m}$Tc-DTPA radiolabelled stealth® liposomal doxorubicin (Caelyx®) in 10 patients with metastatic brain tumours and five patients with brain glioblastoma undergoing radiotherapy. Patients with metastatic brain lesions were treated with 10 consecutive fractions of radiotherapy (whole brain, 3 Gy/fraction, day 1–12) followed by a booster dose of 9 Gy (3 Gy/fraction, day 21–23). Caelyx®, at a dose of 25 mg mg$^{-2}$ was given on day 1 and on day 21. Radiolabelled Caelyx® accumulation was 13–19 times higher in the glioblastomas and 7–13 times higher in the metastatic lesions, as compared to the normal brain. The drug accumulation in the tumoural areas was 40–60% of the accumulation in the bone marrow of the skull bones. The normal brain radioactivity was <4% of the bone marrow, confirming an important shielding effect of the blood–brain barrier in the normal but not in the tumoural tissue. Four of 10 patients with metastatic lesions showed a complete response in CT-scan performed 2 months following therapy. There was no severe toxicity related to radiotherapy or to chemotherapy noted. It is concluded that stealth® liposomal drugs selectively overcome the blood–brain barrier in the tumoural areas. The clinical importance of this observation is now under investigation. © 2000 Cancer Research Campaign

**Keywords**: brain metastasis; glioblastoma; stealth liposomal doxorubicin; radiotherapy

Brain glioblastoma remains an incurable disease. The median survival following surgery is less than 4 months, while additional radiotherapy slightly improves the survival figures (Miller et al, 1990; Davis et al, 1998; Nakagawa et al, 1998). Hyperfractionated and/or accelerated radiotherapy with or without chemotherapy may be of value (Fine et al, 1993; Nelson et al, 1993). The blood–brain barrier is considered a major obstacle for chemotherapeutic drugs to efficiently reach the tumour. Exposure of glioblastoma cells to a higher concentration of drugs may be achieved using compounds that disrupt the blood–brain barrier, such as mannitol (Gumerlock et al, 1992; Doolittle et al, 1998). The inadequate concentration of chemotherapeutic drugs in metastatic brain tumours is also related to this barrier and indeed, radiotherapy alone is the standard modality for the treatment of central nervous system metastasis, the systemic chemotherapy being not effective even for chemosensitive tumours (Jayson and Howell, 1996).

Doxorubicin is one of the most effective drugs for the two principal sources of brain metastasis, namely breast and lung cancer (Valdivieso et al, 1984; Paridaens, 1998). In vitro data show that glioblastoma cell lines are also sensitive to doxorubicin, although overexpression of multidrug-resistance related proteins may counteract the efficacy of the drug (Abe et al, 1994; Stan et al, 1999). The radiosensitizing effects of anthracyclines are well known (Sherman et al, 1982; Bonner and Lawrence, 1990). Recently, a novel formulation of doxorubicin, the so-called Stealth® liposomal doxorubicin (Caelyx®) entered the clinical practice. Stealth® liposomal technology allows a prolonged circulation half-life time of the encapsulated drugs, the liposome size and structure being a major obstacle for extravasation (Gabizon et al, 1994). In that way, the carried drug is selectively accumulated in tissues with increased vascular permeability, such as tumoural tissue (Working et al, 1994). In vivo studies show that liposomes are indeed highly accumulated in experimental gliomas (Shibata et al, 1990; Siegal et al, 1995) suggesting that liposomal drugs may effectively overcome the blood–brain barrier. On the other hand, liposomal doxorubicin, unlike the free doxorubicin, is equally effective in cells with or without multidrug resistance protein expression (Rahman et al, 1992), showing that liposomal technology may also be useful in overcoming inherent resistance of cancer cells to doxorubicin and to other drug substrates of p-glycoprotein efflux pump activity (Warren et al, 1992).

In the present study we investigated the accumulation of radiolabelled stealth® liposomal doxorubicin in patients with glioblastomas and with metastatic brain tumours undergoing radiotherapy. A high intratumoural accumulation is reported, which renders liposomal doxorubicin a candidate for chemo-radiotherapy combinations.

*Received 12 October 1999*
*Revised 19 June 2000*
*Accepted 8 July 2000*

*Correspondence to:* MI Koukourakis

### PATIENTS AND METHODS

Ten patients with brain metastasis entered a phase I/II study of radiotherapy and concomitant administration of stealth® liposomal doxorubicin (Caelyx®; ALZA Corporation, Palo Alto CA, USA). The aims of the study were to investigate the relative accumulation of the radiolabelled Caelyx® in tumour and normal brain areas, as well as the feasibility of Caelyx® combination with brain irradiation. Five more patients with histologically diagnosed brain glioblastoma, treated in an ongoing dose-escalation phase I/II Caelyx®-based chemo-radiotherapy protocol, were also assessed for Caelyx® distribution.

### Recruitment criteria

The criteria for inclusion into the therapeutic protocol were patients with histologically confirmed cancer and limited brain metastatic disease (≤ two lesions), assessed with and measurable on CT-scan or MRI, following typical neurologic symptomatology. Patients with performance status >2. were excluded. Patients with white blood cells <2500 $\mu l^{-1}$, platelets <120 000 $\mu l^{-1}$ or haemoglobin <10 $g/dl^{-1}$ were excluded. Pregnant women or patients with major heart, lung, liver, renal or psychiatric disease, or with haematological malignancies, were excluded. Written informed consent was obtained from all patients.

### Caelyx® radiolabelling and scintigraphic imaging procedure

Five mg of Caelyx® (2.5 ml from the commercially available formulation) were incubated with 20 mCi of $^{99m}$Tc-DTPA for 20 min. The labelling efficiency and the radiochemical purity of the $^{99m}$Tc-DTPA-labelled Caelyx® was determined by instant thin-layer chromatography (ITLC) on 2 × 20 cm chromatography strips as previously described (Koukourakis et al, 1999). Briefly, for the labeling method employed for Caelyx®, ITLC indicated a labelling efficiency of 80%. The main labelled species migrate with the solvent front of the second solvent, while about 19% of the radioactivity, considered as reduced uncomplexed $^{99m}$Tc ($O_2$), remains at the origin. Less than 1% of the radioactivity, corresponding to free pertechnate $^{99m}$Tc($O_4$), migrates with the solvent front of the first solvent.

Radiolabelled Caelyx® was thereafter diluted in 50 ml dextrose water (DW) and was given as a 5 min infusion before the first fraction of radiotherapy. Two hours later, the patient underwent planar (5 min) and SPECT (20 min) scintigraphy using a single-head SPECT camera. Standard regions of interest (ROIs) were drawn on the SPECT images in both the tumour and the corresponding normal brain region of the contralateral hemisphere. Equal ROIs were also drawn on the skull areas. These areas show the accumulation of the liposomal drug in the bone marrow of the skull bones.

### Caelyx® chemotherapy

Following the scintigraphy, patients with metastatic brain tumours were transferred to the chemotherapy unit and an additional dose of 20 mg $m^2$ diluted in 250 ml DW was infused for 30 min. The first fraction of radiotherapy was given 1 h after the Caelyx® infusion. An additional dose of 25 mg $m^{-2}$ of Caelyx® was given on day 21 (when booster fields of radiotherapy were to be treated). Patients with glioblastoma were recruited in an ongoing phase I therapeutic protocol of chemoradiotherapy with Caelyx®-based chemotherapy.

### Radiotherapy for brain metastases

Patients with brain metastases received whole-brain irradiation of 30 Gy (10 fractions of 3 Gy, five fractions per week; days 1–12). An additional dose of 9 Gy (three consecutive fractions of 3 Gy) was given, starting on day 21, with multiple booster fields directed to the metastatic lesions.

### Toxicity evaluation

The toxicity to both chemotherapy and radiotherapy was recorded clinically twice a week, while whole blood cell count, serum urea and creatinine, and liver enzymes were analysed every 2 weeks during the radiotherapy period and for 4 weeks thereafter. Deterioration of the symptomatology during therapy was to be immediately followed by MRI scan to assess whether there was intra-tumoural haemorrhage or increased brain oedema. The World Health Organization toxicity scale was used to record acute toxicity (WHO, 1979). The median follow-up of patients was 6 months (2–12 months).

### Response evaluation of brain metastases

Response to treatment of metastatic brain tumours was assessed with a computed tomography scan (or MRI scan) on days 18–20 (before the booster fields) and 45–60 days following treatment completion. CT scan was done every 2 months for the first 6 months and every 3 months (or earlier if indicated by symptomatology) thereafter. Complete response (CR) was defined as the disappearance of all measurable lesions within 2 months following treatment completion, lasting for at least 2 months after response documentation. Partial response (PR) was defined as a 50–95% reduction in tumour size, while all other cases were considered as non-responders (NR).

### RESULTS

### Drug distribution

Planar and SPECT scintigraphy revealed an intense accumulation of the drug in the tumours for all 15 cases examined (six non-small cell lung carcinomas, one colorectal, three breast carcinomas and five glioblastomas). Figures 1A and 1B show the $^{99m}$Tc-DTPA–Caelyx® SPECT image and the related MRI tomogram respectively, of a small metastatic lesion from a lung adenocarcinoma to the thalamus invading the third ventricle. Despite the small dimensions, an intense accumulation of the radiolabelled Caelyx® is noted. Figures 1C and 1D show the SPECT and the CT-scan image respectively of a glioblastoma of the temporal lobe.

Table 1 shows the total counts from ROIs and count ratios obtained from each analysed case. Overall, the tumour to normal brain ratio was 16 ± 3 and 10 ± 3 for glioblastoma and metastatic tumours, respectively. The drug accumulation was significantly higher in glioblastomas ($P = 0.01$). These results show that stealth liposomal doxorubicin accumulation is 13–19 times higher in the glioblastoma and 7–13 times higher in metastatic tumours, as compared to the normal brain tissue.

© 2000 Cancer Research Campaign

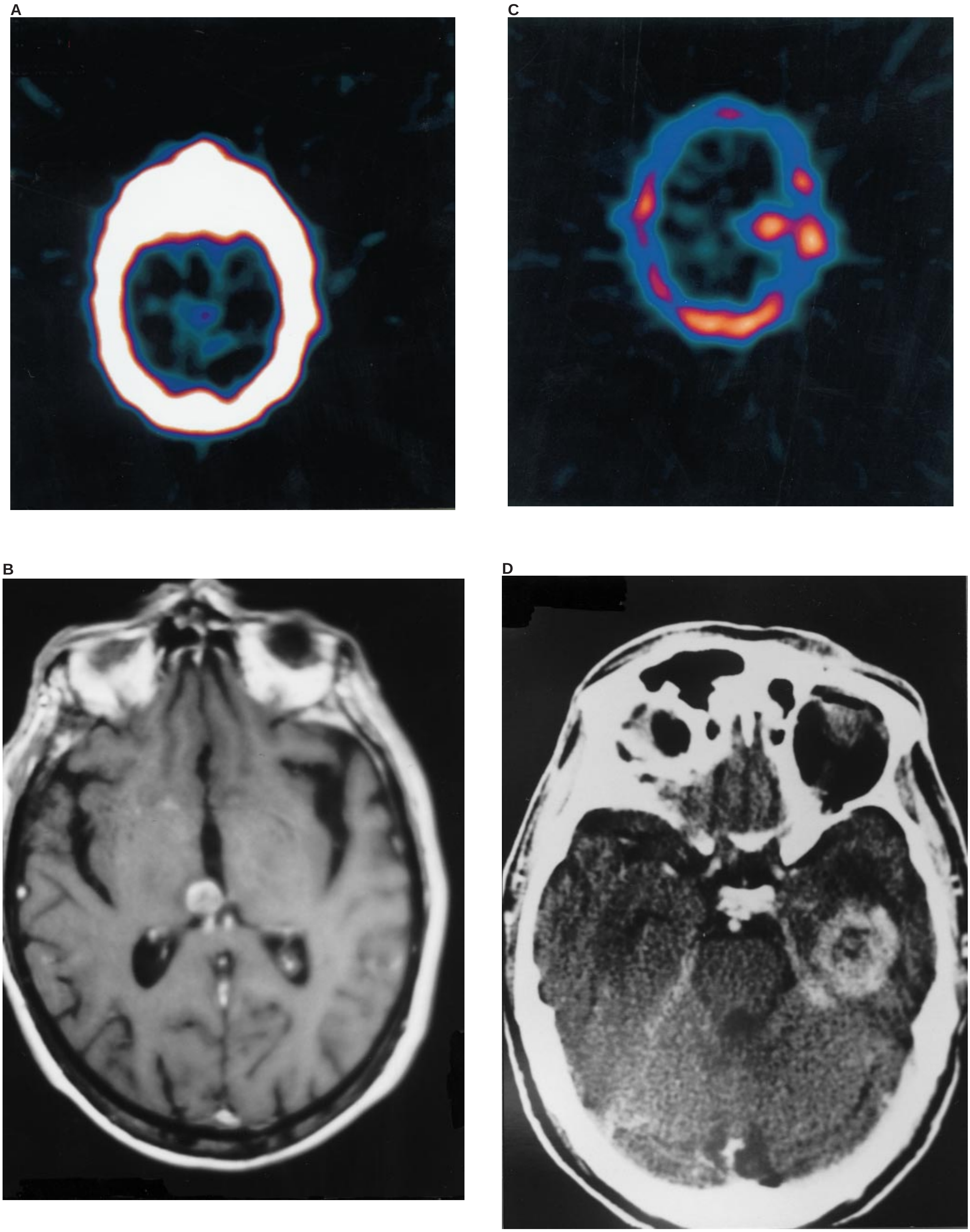

**Figure 1** $^{99m}$Tc-DTPA-Caelyx® SPECT image and the related MRI tomogram of a small metastatic lesion from a lung adenocarcinoma to the thalamus (**A** and **B** respectively). SPECT and the CT-scan image of a glioblastoma of the temporal lobe (**C** and **D** respectively)

*© 2000 Cancer Research Campaign*

**Table 1** Total counts in standard regions of interest (ROIs) and count ratios obtained in brain tumour (T), normal brain (NB) and bone marrow of the skull bones (BM), following intravenous injection of 5 mg of $^{99m}$-Tc-DTPA radiolabelled stealth liposomal doxorubicin

| Patient No/ Disease | Counts in ROIs | | | Count ratio | | |
|---|---|---|---|---|---|---|
| | T | NB | BM | T/NB | T/BM | NB/BM |
| Glioblastoma (location) | | | | | | |
| 1 temporal | 39 | 2.5 | 83 | 15.6 | 0.47 | 0.03 |
| 2 occipital | 29 | 2.4 | 48 | 12.0 | 0.60 | 0.05 |
| 3 frontal/temporal | 42 | 1.9 | 55 | 22.1 | 0.76 | 0.03 |
| 4 frontal | 32 | 2.1 | 54 | 15.2 | 0.59 | 0.03 |
| 5 frontal/temporal | 37 | 2.4 | 62 | 15.4 | 0.59 | 0.03 |
| Overall (mean ± SD) | 35 ± 5 | 2.2 ± 0.2 | 60 ± 13 | 16 ± 3 | 0.6 ± 0.1 | 0.03 ± 0.001 |
| Brain metastasis (primary) | | | | | | |
| 1 NSCLC | 18 | 2.0 | 56 | 9.0 | 0.32 | 0.03 |
| 2 NSCLC | 32 | 2.4 | 42 | 13.3 | 0.76 | 0.05 |
| 3 NSCLC | 19 | 2.2 | 29 | 8.6 | 0.65 | 0.07 |
| 4 NSCLC | 31 | 2.6 | 52 | 11.9 | 0.59 | 0.05 |
| 5 NSCLC | 26 | 2.2 | 62 | 11.8 | 0.41 | 0.03 |
| 6 NSCLC | 15 | 2.0 | 45 | 7.5 | 0.33 | 0.04 |
| 7 Colorectal | 8 | 2.6 | 47 | 3.0 | 0.17 | 0.05 |
| 8 Breast | 30 | 1.9 | 61 | 15.7 | 0.49 | 0.03 |
| 9 Breast | 33 | 2.3 | 59 | 14.3 | 0.55 | 0.04 |
| 10 Breast | 29 | 2.6 | 58 | 11.1 | 0.50 | 0.04 |
| Overall (mean ± SD) | 24 ± 8 | 2.2 ± 0.2 | 51 ± 10 | 10 ± 3 | 0.4 ± 0.1 | 0.04 ± 0.004 |

The drug accumulation in the normal brain was less than 4% of the drug accumulated in the bone marrow, showing a dramatic shielding effect of the blood–brain barrier. On the contrary, the stealth liposomal doxorubicin accumulation in the tumoural areas was very close to the bone marrow accumulation (60% and 40% of the concentration assessed in the bone marrow, for glioblastomas and metastatic tumours, respectively).

### Response and toxicity in patients treated for metastatic brain disease

Complete response was documented in four of 10 of patients (two breast and two non-small cell lung carcinomas) two months following the end of chemo-radiotherapy. Another four patients (one breast and three non-small cell lung carcinomas) showed partial response. There was no haematological toxicity related to Caelyx®. No mucositis was noted. One patient complained of dry palmar skin desquamation but no severe erythrodysestesia was noted in any of the patients. Radiotherapy was accomplished without any increase of early reactions from the irradiated skin and there was no acute neurological toxicity noted. Up to now (median follow-up 6 months) no patient with delayed reactions, such as demyelination or brain necrosis, has been observed.

### DISCUSSION

Pegylated liposomes bear a tightly packed semisolid phospholipid layer and are coated with short polyethylene glycol chains. This formulation enables liposomes to escape from macrophage phagocytosis (Lasic et al, 1991) and to circulate with a median half-life of 45–55 h. Stealth liposomes, being larger than erythrocytes, tend to accumulate in tumoural tissues, since a leaky vascularization allows the liposome extravasation into the stroma (Dvorak et al, 1988). Prolonged circulation of the drug results in continuous selective accumulation in tumours as compared to normal tissues (Stewart and Harrington, 1997). Following their extravasation physico-chemical destabilization and subsequent breakdown of the liposomal envelope is effected as a result of a constellation of local conditions, such as the low pH (Sakayama et al, 1994), the presence of lipases released from dying neoplastic cells (Warren et al, 1992) and also the release of various enzymes or oxidizing molecules and radicals by tumour-infiltrating inflammatory cells (Cobbs et al, 1995; Koukourakis et al, 1998). The cytotoxic effect of radiotherapy may therefore further contribute to the establishment of intra-tumoural conditions that rapidly break down the extravasated liposomes. Stealth liposomal drug combination with radiotherapy is therefore founded on a strong theoretical background. Indeed, experimental studies show a substantial enhancement of the efficacy of fractionated radiotherapy by stealth liposomal drugs including doxorubin (Harrington et al, 1998).

Although certain very chemosensitive tumours that are metastatic to the brain (such as gestational tumours) can be cured with chemotherapy, the overall response rates of metastatic brain tumours are lower than the ones documented for the primary lesions of origin. This shows that, apart from the chemoresistance related to the cancer cell biology itself, the blood–brain barrier may further reduce the efficacy of chemotherapy by inhibiting the achievement of adequate drug concentrations in brain tumours. Several experimental studies suggest that liposomal technology may be of importance in overcoming the blood–brain barrier. Shibata et al (1990) reported a study where liposomes containing horse-radish peroxidase were injected in glioblastoma-bearing rats. Histochemical staining showed that 30 min following injection the liposomes were highly accumulated in and around the tumour endothelium, but also within tumour cells (Shibata et al, 1990). In another study, Siegal et al (1995) also reported an up to 30-fold higher accumulation of stealth liposomal doxorubicin in the cerebrospinal fluid of rats bearing an inoculated to the brain sarcoma as compared to the concentrations achieved with free doxorubicin. Liposome-encapsulated taxol was also found to be

 © 2000 Cancer Research Campaign more effective than free taxol in two brain tumours xenografted into nude mice (Riondel et al, 1992). More recently, Khalifa et al (1997) examined the distribution of $^{111}$In-labelled liposomes in patients with glioblastoma, showing a remarkable increased accumulation in the tumours as compared to normal brain. This differential accumulation rate was increasing even 72 h following the administration of liposomes.

The present study confirms an intense accumulation of radiolabelled $^{99m}$Tc-DTPA–Caelyx® in patients with glioblastomas and metastatic tumours of the brain. The tumour to normal brain count ratio showed a 7–19-fold higher accumulation of the drug in the tumoural as compared to the normal brain tissue. The drug concentration in the tumour was 40–60% of that recorded in the bone marrow, an area where liposomes are highly accumulated (Gabizon et al, 1994). The blood–brain barrier shielding effect was confirmed in the normal brain tissue, where the drug accumulation was less than 4% of that noted in the bone marrow.

Another observation was the higher accumulation of the drug in glioblastomas as compared to the metastatic tumours. This may be explained by pathological studies showing that glioblastomas are among the tumours of highest vascularization (Plate and Risau, 1995). Indeed in a previous study of ours, highly vascularized NSCLC showed a higher accumulation of radiolabelled Caelyx® (Koukourakis et al, 1999). Highly angiogenic gliomas are also associated with a worse prognosis (Leon et al, 1996). Combination of radiotherapy with drugs the accumulation of which depends on the vascular density, may therefore be important for the treatment of highly angiogenic gliomas.

It is concluded that stealth liposomal drugs selectively overcome the blood–brain barrier in the tumoural areas. The 7–19-fold higher accumulation of stealth liposomal doxorubicin in both glioblastomas and metastatic tumours of the brain strongly suggests that Caelyx® may increase the effectiveness of radiotherapy for brain tumours. Clinical studies are ongoing to establish a well tolerated, high-dose intensity schedule of Caelyx® chemoradiotherapy and to assess eventual benefit from such a regimen.

### REFERENCES

Abe T, Hasegawa S, Taniguchi K, Yokomizo A, Kuwano T, Ono M, Mori T, Hori S, Kohno K and Kuwano M (1994) Possible involvement of multidrug-resistance-associated protein (MRP) gene expression in spontaneous drug resistance to vincristine, etoposide and adriamycin in human glioma cells. *Int J Cancer* **58**: 860–864

Bonner JA and Lawrence TS (1990) Doxorubicin decreases the repair of radiation-induced DNA damage. *Int J Radiat Biol* **57**: 55–64

Cobbs CS, Brenman JE, Aldape KD, Bredt DS and Israel MA (1995) Expression of nitric oxide synthase in human central nervous system tumors. *Cancer Res* **15**: 727–730

Davis FG, Freels S, Grutsch J, Barlas S and Brem S (1998) Survival rates in patients with primary malignant brain tumors stratified by patient age and tumor histological type: an analysis based on surveillance, epidemiology, and end results (SEER) data, 1973–1991. *J Neurosurg* **88**: 1–10

Doolittle ND, Petrillo A, Bell S, Cummings P and Eriksen S (1998) Blood–brain barrier disruption for the treatment of malignant brain tumors: The National Program. *J Neurosci Nurs* **30**: 81–90

Dvorak HF, Nagy JA, Dvorak JT and Dvorak AM (1988) Identification and characterization of the blood vessels of solid tumors that are leaky to circulating macromolecules. *Am J Pathol* **133**: 95–109

Fine HA, Dear KB, Loeffler JS, Black PM and Canellos GP (1993) Meta-analysis of radiation therapy with and without adjuvant chemotherapy for malignant gliomas in adults [see comments]. *Cancer* **71**: 2585–2597

Gabizon A, Catane R, Uziely B, Kaufman B, Safra T, Cohen R, Martin F, Huang A, Barenholz Y (1994) Prolonged circulation time and enhanced accumulation in malignant exudates of doxorubicin encapsulated in polyethylene-glycol coated liposomes. *Cancer Res* **54**: 987–992

Gumerlock MK, Belshe BD, Madsen R and Watts C (1992) Osmotic blood–brain barrier disruption and chemotherapy in the treatment of high grade malignant glioma: patient series and literature review. *J Neurooncol* **12**: 33–46

Harrington KJ, Rowlinson-Busza G, Uster PS, Whittaker J and Stewart JSW (1998) Stealth® liposome encapsulated doxorubicin (SLED) and iododeoxyruridine (SLIIUDR) as radiation sensitizers in head and neck squamous cell cancer xenograft tumors (HNSCCXT). *Proc Am Soc Clin Oncol* **17**: 230a (abst 881)

Jayson GC and Howell A (1996) Carcinomatous meningitis in solid tumours. *Ann Oncol* **7**: 773–786

Khalifa A, Dodds D, Rampling R, Paterson J and Murray T (1997) Lipsomal distribution in malignant glioma: possibilities for therapy. *Nucl Med Commun* **18**: 17–23

Koukourakis MI, Giatromanolaki A, Kakolyris S, O'Byrne KJ, Apostolikas N, Skarlatos J, Gatter K, and Harris AL (1998) Different patterns of stromal and cancer cell thymidine phosphorylase reactivity in non-small cell lung cancer. Impact on neoangiogenesis and survival. *Br J Cancer* **77**: 1696–1703

Koukourakis MI, Koukouraki S, Giatromanolaki A, Archimandritis SC, Skarlatos J, Beroukas K, Bizakis J, Retalis G, Karkavitsas N and Helidonis ES (1999) Stealth liposomal doxorubicin and conventionally fractionated radiotherapy in the treatment of locally advanced non-small cell lung and head and neck cancer. *J Clin Oncol* **17**: 3512–3521

Lasic DD, Martin FJ, Gabizon A, Huang SK and Papahadjopoulos D (1991) Sterically stabilized liposomes: a hypothesis on the molecular origin of the extended circulation times. *Biochim Biophys Acta* **1070**: 187–192

Leon SP, Folkerth RD and Black-PM (1996) Microvessel density is a prognostic indicator for patients with astroglial brain tumors. *Cancer* **77**: 362–372

Miller PJ, Hassanein RS, Giri PG, Kimler BF, O'Boynick P and Evans RG (1990) Univariate and multivariate statistical analysis of high-grade gliomas: the relationship of radiation dose and other prognostic factors. *Int J Radiat Oncol Biol Phys* **19**: 275–280

Nakagawa K, Aoki Y, Fujimaki T, Tago M, Terahara A, Karasawa K, Sakata K, Sasaki Y, Matsutani M and Akanuma A (1998) High-dose conformal radiotherapy influenced the pattern of failure but did not improve survival in glioblastoma multiforme. *Int J Radiat Oncol Biol Phys* **40**: 1141–1149

Nelson DF, Curran WJ Jr, Scott C, Nelson JS, Weinstein AS, Ahmad K, Constine LS, Murray K, Powlis WD, Mohiuddin M (1993) Hyperfractionated radiation therapy and bis-chlorethyl nitrosourea in the treatment of malignant glioma – possible advantage observed at 72.0 Gy in 1.2 Gy BID fractions: report of the Radiation Therapy Oncology Group Protocol 8302. *Int J Radiat Oncol Biol Phys* **25**: 193–207

Paridaens R (1998) Efficacy of paclitaxel or doxorubicin used as single agents in advanced breast cancer: a literature survey. *Semin Oncol* **25**(Suppl 12): 3–6

Plate KH and Risau W (1995) Angiogenesis in malignant gliomas. *Glia* **15**: 339–347

Rahman A, Husain SR, Siddiqui J, Verma M, Agresti M, Center M, Safa AR and Glazer RI (1992) Liposome-mediated modulation of multidrug resistance in human HL-60 leukemia cells. *J Natl Cancer Inst* **84**: 1909–1915

Riondel J, Jacrot M, Fessi H, Puisieux , and Potier (1992) Effects of free and liposome-encapsulated taxol on two brain tumors xenografted into nude mice. *In Vivo* **6**: 23–27

Sakayama K, Masuno H, Miyazaki T, Okumura H, Shibata T and Okuda H (1994) Existence of lipoprotein lipase in human sarcomas and carcinomas. *Jpn J Cancer Res* **85**: 515–521

Sherman DM, Carabell SC, Belli JA and Hellman S (1982) The effect of dose rate and adriamycin on the tolerance of thoracic radiation in mice. *Int J Radiat Oncol Biol Phys* **8**: 45–51

Shibata S, Ochi A and Mori K (1990) Liposome as carriers of cisplatin into central nervous system – experiments with 9L gliomas in rats. *Neurol Med Chir* **30**: 242–245

Siegal T, Horowitz A and Gabizon A (1995) Doxorubicin encapsulated in sterically stabilized liposomes for the treatment of a brain tumor model: biosistribution and therapeutic efficacy. *J Neurosurg* **83**: 1029–1037

Stan AC, Casares S, Radu D, Walter GF and Brumeanu TD (1999) Doxorubicin-induced cell death in highly invasive human gliomas. *Anticancer Res* **19**: 941–950

Stewart S and Harrington KJ (1997) The biodistribution and pharmacokinetics of stealth liposomes in patients with solid tumors. *Oncology* **10** (Suppl 11): 33–37

Valdivieso M, Burgess MA, Ewer MS, Mackay B, Wallace S, Benjamin RS, Ali MK, Bodey GP and Freireich EJ (1984) Increased therapeutic index of weekly doxorubicin in the therapy of non-small cell lung cancer: a prospective, randomized study. *J Clin Oncol* **2**: 207–214

© 2000 Cancer Research Campaign 

Warren L, Jardillier JC, Malrska A and Akeli MG (1992) Increased accumulation of drugs in multidrug-resistant cells induced by liposomes. *Cancer Res* **52**: 3241–3245

Working PK, Newsman MS, Huang SK, Mayhew E, Vaage J and Lasic DD (1994) Pharmacokinetics, biodistribution and therapeutic efficacy of doxorubicin encapsulated in stealth® liposomes (Doxil®). *J Liposom Res* **4**: 667–687

World Health Organization (1979) *Handbook for reporting results of cancer treatment*. WHO Offset Publications: Geneva

 *© 2000 Cancer Research Campaign*
